# Importance of Oceanian small mountainous rivers (SMRs) in global land-to-ocean output of lignin and modern biospheric carbon

**DOI:** 10.1038/srep16217

**Published:** 2015-11-20

**Authors:** Hongyan Bao, Tsung-Yu Lee, Jr-Chuan Huang, Xiaojuan Feng, Minhan Dai, Shuh-Ji Kao

**Affiliations:** 1 State Key Laboratory of Marine Environmental Science, Xiamen University, 361102, Xiamen, China; 2 Department of Geography, National Taiwan Normal University, Taipei, Taiwan; 3 Department of Geography, National Taiwan University, Taipei, Taiwan; 4 State Key Laboratory of Vegetation and Environmental Change, Institute of Botany, Chinese Academy of Sciences, Beijing, China

## Abstract

The land-to-ocean export of particulate organic carbon (POC) connects carbon flow from the atmosphere through land to the ocean, of which the contemporary fraction that reaches the deep sea for burial may effectively affect atmospheric CO_2_. In this regard, small mountainous rivers (SMRs) in Oceania, a global erosion hotspot driven by torrential typhoon rain and active earthquakes are potentially important. Here we measured typhoon lignin discharges for Taiwan SMRs. We found that the particulate lignin export in 96 hours by a single SMR amounting to ~20% of the annual export by Mississippi River. The yearly particulate lignin discharge from Taiwan Island (35,980 km^2^) is governed by the frequency and magnitude of typhoon; thus, the historical lignin export ranged widely from 1.5 to 99.7 Gg yr^−1^, which resulted in a 10–100 times higher areal yield relative to non-Oceanian rivers. The lignin-derived modern POC output from Oceania region is 37 ± 21 Tg C yr^−1^, account for approximately 20% of the annual modern POC export from global rivers. Coupled with the hyperpycnal pathway, the forested watersheds of SMRs in Oceania may serve as a giant factory to rapidly produce and efficiently convey modern POC into deep sea for sequestration.

The transfer of terrestrial POC from land to the ocean represents a critical linkage in global carbon cycle^1^. This process is particularly important in small river watersheds of high-standing islands in the Oceanian region across the western Pacific as characterized by high terrestrial production, frequent landslides induced by typhoon and active earthquakes^2^,^3^,^4^,^5^,^6^. In contrast to large rivers, small mountainous watersheds have insufficient capacity to store eroded sediments and soils in the floodplain. The abundant supply and shorter residence time of eroded material in watersheds of Oceania result in a disproportionately high discharges of terrestrial material including particulate organic carbon and metals into the ocean^7^,^8^,^9^,^10^,^11^. Moreover, the extreme rainfall event usually causes high turbidity (sediment concentration >40 g L^−1^) flood, thus, generates hyperpycnal flow to transfer terrestrial POC episodically and efficiently into the deep sea for burial^12^,^13^,^14^,^15^. Despite the importance of episodic extreme events, flood monitoring, particularly for biomarkers such as lignin, had rarely been conducted, because of their unpredictable occurrence and the monitoring can be both costly and physically dangerous.

Typhoon-triggered deep landslides supply clastic sediments and organic materials, in which fossil POC from rock and biospheric POC were mixed together^8^,^16^,^17^ (hereafter named as total POC, tPOC). Marine burial of biospheric POC represents a major C sink over geological timescales. However, biospheric POC export from rivers contain aged POC associated with mineral soils^18^ and recently-synthesized terrigenous POC^3^ from higher plants. In the contemporary carbon cycle, the reallocation of fossil and aged biospheric POC from soil pool to marine sediment pool is ineffective or less active C sink. Oppositely, the oxidation of fossil organic carbon served as a C source^19^. To better quantify the transfer of active carbon from land and ocean, radiocarbon-based two end-member mixing model was usually applied to differentiate modern from fossil OC^3^,^17^,^20^,^21^,^22^,^23^. Based on these studies, Oceanian SMRs discharge ~11–40 Tg C yr^−1^ of modern terrigenous POC^17^. However, two end-member might not be sufficient since pre-aged POC that includes refractory terrigenous POC pool from mineral soil^18^,^24^ may contribute a significant fraction to the total POC export from rivers (e.g., Ganges–Brahmaputra)^18^,^24^,^25^. Here in this paper, we do not argue the necessity to include the pre-aged organic carbon in two end-member mixing model, alternatively, we aims to provide an independent approach to quantify the export of recently-photosynthesized POC not only for episodic events in Taiwan but also scale up to Oceania to discuss its potential role in global carbon cycle.

As a unique and the second most abundant biopolymer (representing approximately 30% of biosynthesized organic carbon^26^) in vascular plants, lignin has been widely used in tracing terrigenous POC in rivers and oceans^27^,^28^. 70–80% of lignin are distributed in the surface layers of soils (O and A horizons)^29^, which are mainly plant litters as well as mineral soil that is rich in organic carbon^30^,^31^,^32^, representing modern POC. The lignin in soil profiles decreases rapidly^30^,^31^ and its ratio to tPOC can be more than 10 times higher in surface soils than in deeper soils^29^, which indicates that lignin degraded much faster than bulk soil organic carbon^33^. Recent study of compound-specific radiocarbon dating for lignin phenols on particulate matter in river suggested that lignin represents the relatively young fraction of terrigenous POC during fluvial transport^34^,^35^. Furthermore, the degradation indicator, acid to aldehyde ratio of vanillyl of lignin ((Ad/Al)v), is much higher in deeper soil (~1.5) than that in surface soil (~0.7)^36^; accordingly, the ratio may also serve as an indicator of the source region (surface versus deep soil) of lignin phenols on riverine particle. Those characteristics of lignin in soil profile make it a tool to trace and potentially quantify the freshly produced terrigenous organics (<~200 years)^3^ exported to the sea. Since no ^14^C involves, lignin method avoids the interference from pre-aged carbon.

Here we present a study carried out in the mountainous Taiwan Island. Small mountainous rivers in Taiwan (Supplementary Table S1) are characterized with steep river channel and small basin area^4^. These small mountainous watersheds were rapidly flushed during typhoons, the lag time between flood peak and rain peak is only ~6 hours^12^,^40^ and most of the typhoon events last for only ~3 days^4^. More importantly, most of the sediment and POC output occurred during flood events^3^,^17^. Oceania region shares similar climatic and geomorphic features in Taiwan as indicated in previous studies^38^.

We collected suspended sediments at 3-hour intervals from Jhuoshuei River and Liwu River during Typhoon Mindulle (6:00, 1 July 2004 to 12:00, 4 July 2004) and Morakot (0:00, 7 August 2009 to 21:00, 9 August 2009), respectively (Fig. 1), to evaluate the typhoon-associated lignin discharge. Suspended particles were collected using low-density polyethylene bottle attached to a weighted metal frame that was gradually lowered from the bridge^12^. However, the fast flow (typically >4 m s^−1^)^39^ prohibits the vertical sampling. The sampling over hydrograph improves not only the accuracy of our estimate but also the understanding of terrigenous POC transfer dynamics during episodic events. We also sampled at upstream Jhuoshuei during Typhoon Mindulle and ten other rivers during various typhoons (Typhoon Morakot and Typhoon Doug, Fig. 1, Supplementary Table S1) to construct an empirical relation that can be applied for lignin export from the entire Taiwan. Our approach provides an alternative estimate (^14^C independent) for export of modern terrigenous POC from Oceanian SMRs. To be convincing, we also drew lignin associated statistical data of Taiwan soils (unpublished) for discussion.

## Results and Discussion

### Event export of lignin from Jhuoshuei River and Liwu River

**Figure 2:**
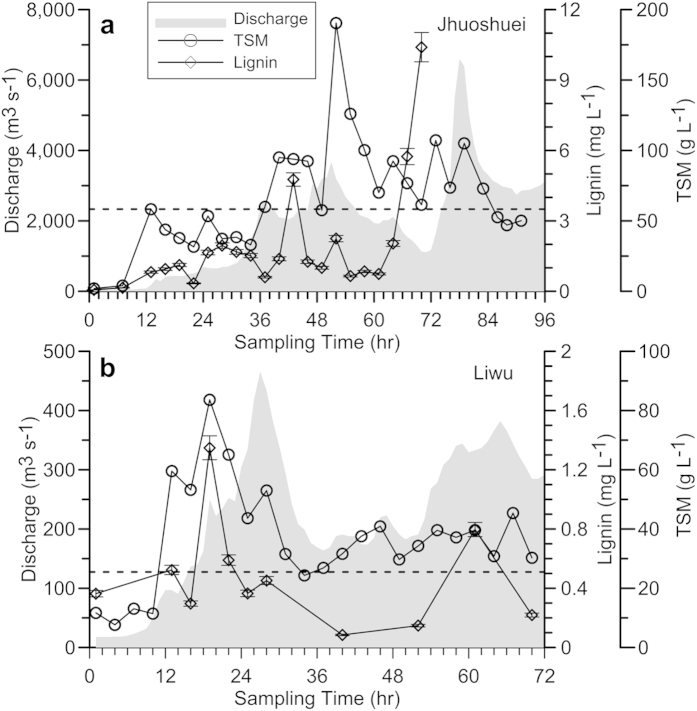
The temporal variation of water discharge, total suspended matter concentration (TSM, g L^−1^) and lignin concentration (Lignin, mg L^−1^) during the typhoon flood. (**a**)Jhuoshuei River during Mindulle. (**b**) Liwu River during Morakot. Dashed lines indicate the discharge-weighted lignin concentrations. Error bars indicate the standard deviation of analytical measurement.

During Typhoon Mindulle at Jhuoshuei, water discharge (Q_w_) increased from 54 m^3^ s^−1^ to 6,390 m^3^ s^−1^ in 78 hours, and the total suspended matter (TSM) concentration increased from 1 g L^−1^ to ∼190 g L^−1^ (Fig. 2a). The concentration of lignin phenols increased from 0.06 mg L^−1^ to 10.4 mg L^−1^, with a discharge-weighted concentration of 3.5 mg L^−1^ (Fig. 2a). The concentration of lignin (in mg L^−1^) showed a significant positive correlation with Q_w_ (r = 0.85, *p *< 0.001, n = 22). Similar positive relations have also been observed in some North American SMRs^6^, suggesting that torrential rain and subsequent water discharge exacerbates both the catchment supply and riverine export of lignin. The lignin/tPOC ratio ranged from 1.5 to 23.7 mg (g tPOC)^−1^ during entire monitoring period (data not shown). The highest value occurred during the discharge peak approaching the mean lignin/tPOC in the surface soil of Jhuoshuei basin (38 ± 26 mg (g OC)^−1^, n=21, depth <20 cm, unpublished data). Such resemblance in lignin/tPOC ratio during peak discharge affirms again the role of rainfall in remobilization of modern biospheric POC^3^.

A significant power correlation was obtained between Q_lignin_ and Q_w_ (Log Q_lignin_= 1.729 Log Q_w_ –2.194, R^2^=0.92, p<0.001, n = 22). Basing on this empirical correlation, lignin flux could be estimated during Typhoon Mindulle by substituting continuously measured water discharge rate (see Methods). The export of particulate lignin during Mindulle (96 hours) was estimated to be 2.2 ± 0.5 Gg. Together with the consecutive Typhoon Aere (96 hours, Supplementary Fig. S1), the two events discharged 4.0 ± 0.9 Gg of particulate lignin from Jhuoshuei. Such short-lived event flux is as high as one third of the annual particulate lignin export from Changjiang (14.9 Gg yr^−1^)^40^ and Mississippi River (13 Gg yr^−1^)^41^, both hold ~600 times larger catchment area relative to Jhuoshuei. The areal export of lignin in hourly basis (kg lignin km^−2^ hr^−1^) from Jhuoshuei River during one single event is hence more than 16,900 times higher than that of Mississippi River^41^. The disproportionately high lignin discharge in such extreme event has never been reported yet. Our observation in Taiwan highlights the importance of rarely-observed cyclone-driven export of lignin from Oceanian SMRs. Note that 2004 is not a unique year in Jhuoshuei’s history since the sediment load in 2004 (108 Mt) ranks only at the middle level in the wide spectrum of annual sediment load (1970–2010, ranging from 2.8–263.8 Mt yr^−1^; Supplementary Fig. S1). The huge variability in Jhuoshuei’s annual sediment load implies that annual lignin export may also be highly variable depending upon the frequency and magnitude of rainfall driven by landfall typhoon.

In Liwu River during Typhoon Morakot, the lignin ranged from 0.1 to 1.4 mg L^−1^ with a discharge-weighted concentration of 0.5 mg L^−1^ (Fig. 2b), which is much lower than that observed in Jhuoshuei River (Fig. 2a). Both the TSM and lignin peaked during the rising limb of discharge, implying a channel washing effect. However, lignin concentrations remained low during and after the first discharge peak, implying no significant landslide was triggered. Similar to Jhuoshuei River, lignin flux from Liwu during Typhoon Morakot was also estimated by the event rating curve (Log Q_lignin_ = 1.092 Log Q_w_-0.644, R^2^ = 0.62, p < 0.01, n = 11). According to this relationship, the lignin output by Morakot from Liwu was 0.05 ± 0.01 Gg only. In fact, the peak water discharge in Liwu during our sampling is trivial in Liwu’s historical records (Supplementary Fig. S2). While Typhoon Morakot killed 400 people by eliminating a village and caused ten thousands landslides in southwestern Taiwan^42^. Our monitoring further demonstrates a strong spatial heterogeneity in lignin export that is controlled by the distribution of rainfall foci even within a relatively small island.

### Lignin exports from SMRs

**Figure 3:**
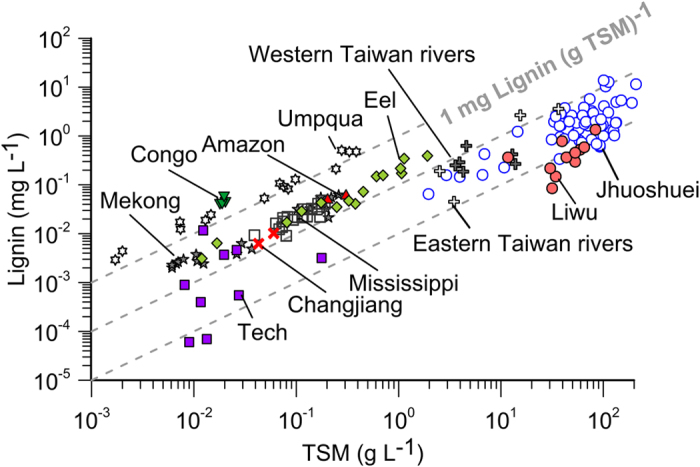
Scatter plot of lignin concentration (mg L^−1^) against TSM (g L^−1^) for Taiwan SMRs and other rivers. Three grey lines are for 0.01, 0.1 and 1 mg Lignin (g TSM)^−1^ line, respectively. Data sources: Taiwan SMRs, this study; Amazon River^51^, Congo River^52^; Changjiang^40^; Mekong River^53^; Mississippi River^41^; Tech River^54^; Eel River and Umpqua River^6^; Oceanian SMRs^55^.

**Figure 4:**
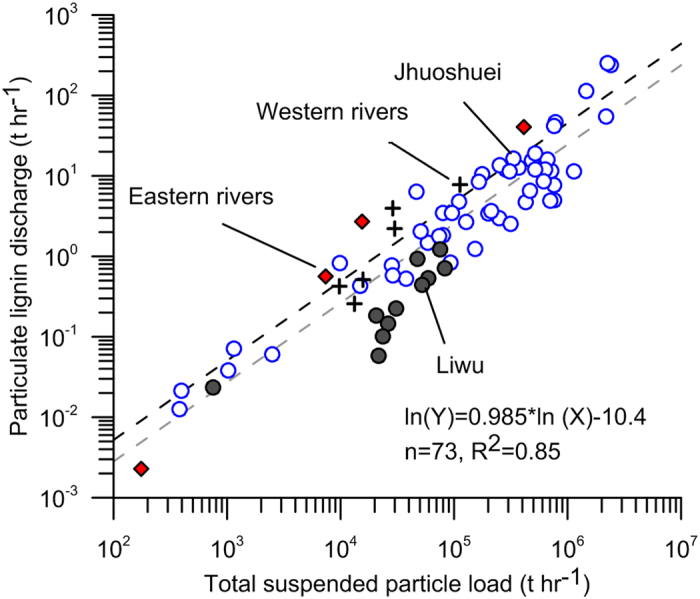
Scatter plot between particulate lignin discharge (t hr^−1^) and total suspended particle load (t hr^−1^) for flood samples island-wide. The gray regression stands for the original log-log linear regression. The black dashed line and the equation are the bias-corrected for back transformation log-log linear regression following Kao *et al.* (2005). The residual is 35% for both over- and under-estimate and applied onto the annual lignin load in Supplementary Fig. S3.

We further compared the particulate lignin concentration against TSM in other ten Taiwan SMRs and documented global rivers (Fig. 3). Both the TSM and lignin concentrations of Taiwan SMRs are on average 2–4 orders of magnitude higher than non-Oceanian rivers. The TSM-normalized lignin concentrations (slopes) of Taiwan SMRs fall within the range of 0.01–0.1 mg lignin (g TSM)^−1^ with a flux-weighted mean of 0.05 mg lignin (g TSM)^−1^, lower than that of other non-Oceanian rivers. The lower lignin content in TSM suggests that lignin is diluted by clastic sediment carried by Taiwan SMRs. Yet, the dilution factor is relatively small when compared with orders of magnitude increase of sediment concentration. Nevertheless, the strong correlation between Q_lignin_ (t hr^−1^) and sediment discharge (Q_s_, t hr^−1^) (Fig. 4) allows us to estimate lignin discharge based on historical sediment loads for all majors rivers in Taiwan. Coupled with the long-term record of daily sediment discharge from 16 Taiwan SMRs (Supplementary Table S2 and Supplementary Fig. S3 for total lignin export from 16 Taiwan SMRs), the annual lignin flux from Taiwan is estimated to be 1.5 Gg yr^−1^ to 99.7 Gg yr^−1^ with a mean of 19.7 Gg yr^−1^ (Table 1). The resulted annual yield of lignin is 0.04–2.77 t km^−2^ yr^−1^ with a mean value of 0.55 t km^−2^ yr^−1^, which is again 10–100 times higher than those reported for large rivers and 18–55 times higher than non-Oceanian SMRs (Table 1).

Similar to Taiwan, many other SMRs in high-standing Oceania (including Papua New Guinea, New Zealand, Philippines, Indonesia and Malaysia) also have high erosion rates^43^, large POC export^10^ and potentially large lignin export. Multiply the above lignin yield (0.55 t km^−2^ yr^−1^) of Taiwan SMRs by the total basin area of 2.7 × 10^6^ km^2^ for entire Oceania, we estimated that the total lignin flux from Oceanian SMRs is 114–7482 Gg yr^−1^, with a mean value of 1476 Gg yr^−1^.

### Lignin-derived modern POC export from Oceanian SMRs

**Figure 5:**
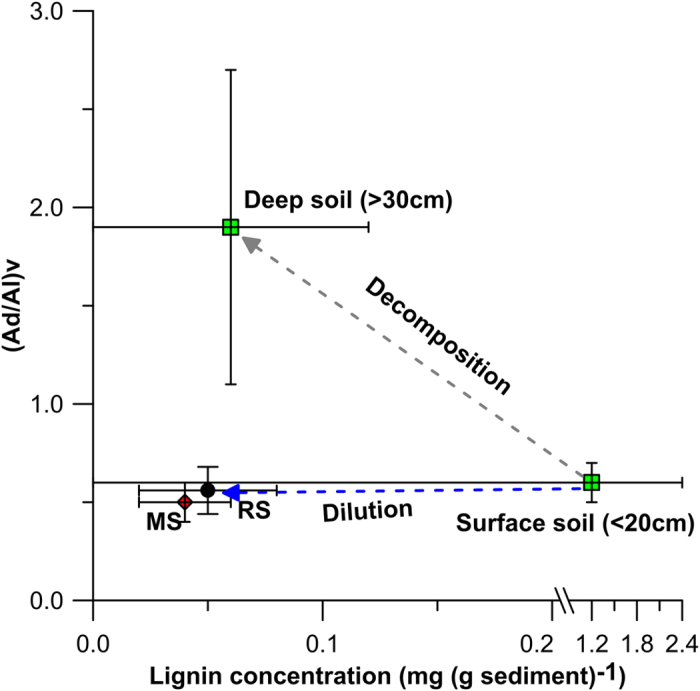
Lignin concentration versus (Ad/Al)v in river suspended particles (RS), surface soil, deep soil and marine sediments (MS), mean ± 1SD are shown. Lignin phenols data for marine sediments are from Kuo *et al.*, 2014^48^.

Since lignin is predominately sourced from surface soil, we added some statistics of unpublished soil data for discussion. The mean of lignin to tPOC ratios for surface soils in Jhuoshuei basin is close to the report for surface soils in another Oceanian SMR basin (Fly River in Papua New Guinea, 43 ± 21 mg (g tPOC)^−1^)^36^. Meanwhile, the lignin/tPOC ratio in Jhuoshuei’s deep soil (10 ± 8 mg (g tPOC)^−1^, n = 14, unpublished data, Fig. 5) was much lower than surface soil. Above facts suggest that lignin phenols are preferentially degraded in soils in comparison to other compounds^29^,^32^, such as hydroxyalkanoic acids^29^. As aforementioned, the lignin/tPOC ratio during the peak flood was approaching that of the surface soil suggesting a greater contribution of lignin from surface soil to the export peak. Since the lag time between rain peak and flood peak is within a day^12^,^40^, which is too short to allow any significant degradation during such ephemeral transport. In fact, the measured (Ad/Al)v values supported this notion (Fig. 5). The observed (Ad/Al)v ratios of all river samples fell within a narrow range (0.6 ± 0.1) being identical to the surface soil (0.7±0.2, depth <20 cm, n = 21, Fig. 5). By contrast, much higher values were observed for deeper soil (1.9 ± 0.8, depth >30 cm, n = 14, Fig. 5). Such constant and highly similar (Ad/Al)v ratio between riverine particle and surface soil implied that degradation of lignin in the river channel during such quick fluvial transport is minimum and the contribution of deep soil to the lignin output is negligible. Here we applied a chart of cumulative flux against flow return time (Supplementary Fig. S4) to illustrate the importance of flood in terms of longer term transport of particulate phases. Take Jhuoshuei as an example, cumulative figure show that approximately 70% of lignin is exported with a flow return time >0.4 years (mean flow return time of Typhoon Mindulle).

The lignin/tPOC ratios in soils are affected by grain size^44^. However, the steep slope of river channel creates fast flow during events (typically >4 m s^−1^)^39^. The fast flow coupled with shallow water depth and rugged bottom generates turbulent water that mixed particles well during transport. How well the mixing is cannot be known since the fast flow prohibits the vertical sampling. Nevertheless, in most Taiwan rivers, mud fraction dominates the suspended load^39^, thus, grain size effect on the lignin/tPOC ratio is unlikely. The large variation in lignin/tPOC ratio (by factor of ~20) along with the hydrography, in fact, was influenced by the addition of lignin-free POC. Therefore, the modern POC export from Taiwan SMRs can be calculated by dividing lignin flux by lignin/tPOC of surface soil, and the result is 0.5 ± 0.3 Tg yr^−1^ (13.9 ± 9.4 t km^−2^ yr^−1^).

On the other hand, Hilton *et al.* (2011, 2012) estimated that tPOC export from Taiwan SMRs is approximately 1.8 Tg yr^−18, ^9. We also estimated the long-term tPOC export using the similar method for lignin phenols (Supplementary Fig. S5), our estimated tPOC flux (1.6 Tg yr^−1^) is very close to previous report. On long-term basis, the lignin-derived modern POC in Taiwan SMRs contribute approximately 31 ± 21% to the tPOC flux being consistent with previous report measured by ^14^C^17^.

Multiply by the total area for entire Oceania, the total modern POC flux from Oceanian SMRs was estimated to be 37 ± 25 Tg yr^−1^. In addition to sediment associated modern POC export, Oceanian SMRs also export modern POC in the form of coarse wood debris, e.g., during Typhoon Morakot, 1.8–4.0 Tg C was estimated to be transported to the oceans by Taiwan SMRs^45^. In the meantime, according to our estimate, Typhoon Morakot delivered approximately 94 ± 33 Gg and 2.5 ± 1.9 Tg of sediment associated lignin and modern POC, respectively. The amount of modern POC delivered by coarse wood is in the similar magnitude to the lignin derived modern POC. Such equal amount of aboveground biomass and lignin-derived POC exports suggested deep erosion (landslide) eliminates entire critical zone of terrestrial forest ecosystem. Nevertheless, most the wood is floating yet sediment associated modern POC is sinking and likely transported into deep sea rapidly.

### Global significance

The mean Oceanian lignin flux (1476 Gg yr^−1^) is substantially higher than the summarized total particulate lignin export from 6 major rivers globally and 12 Arctic Rivers (437 Gg yr^−1^; Table 1). Our results indicate that the export of lignin from Oceanian SMRs is not only significant in areal yield but also in flux.

Our extrapolation for the modern POC export from Oceanian SMRs (37 ± 25 Tg yr^−1^) broadly agrees with previous estimation (11–40 Tg yr^−1^) by the two-end member radiocarbon mixing model^17^. Likely, in high physical erosion watersheds the pre-aged component is less important due to short residence time of soil. According to our results, the modern POC discharged from Oceanian SMRs accounts for approximately 20% of the global biospheric POC output^5^,^46^, yet they only cover 1.8% of the global land surface area, suggesting the importance of Oceanian SMRs in the land-ocean modern POC transfer. Our results also support the erosional control on global carbon export from the terrestrial biosphere^46^.

In large river systems such as Amazon, a large fraction of recent terrigenous POC is degraded during transport^47^, and hence less recent terrigenous POC may reach the ocean, not to mention the deep ocean for burial. In sharp contrast, episodic events conveys efficient transport^17^ of recent organics produced on land to the ocean with minimal degradation during the export from land to river and from river to the ocean in SMRs. The TSM normalized lignin concentration and (Ad/Al)v between river suspended particles (lignin concentration: 0.05 ± 0.03 mg (g sediment)^−1^, (Ad/Al)v: 0.6 ± 0.1)) and surface sediment around Taiwan^48^ (0–10 cm, lignin concentration: 0.04 ± 0.02 mg (g sediment)^−1^, (Ad/Al)v: 0.5 ± 0.1, n = , Fig. 5) are close, further indicate the efficient preservation of mineral associated modern POC in marine environment^17^.

To sustain the luxury vegetation in Oceanian watersheds, a rapid rejuvenation of terrestrial biome is required. Coupled with the hyperpycnal pathway, the forested watersheds of SMRs in the entire Oceania may hence act as a global hotspot to rapidly generate and efficiently convey recent terrigenous POC to the sea for sequestration.

## Methods

### Analysis

Suspended particle was filtered onto pre-combusted GF/F filters^12^, and was then separated from the filters for further analysis. Prior to the measurement of POC concentration, samples were treated with 1N HCl at 60 °C for 16 h to remove carbonate; the residue was then centrifuged and freeze-dried. The POC was determined by Carlo-Erba 2100 elemental analyser connected to a Thermo Finnigan Delta plus. The relative precision is better than 2%.

Lignin phenols were analyzed by CuO oxidation method^49^. About 0.5–1 g of dried and ground samples mixed with CuO, Fe(NH_4_)_2_(SO_4_)_2_ and NaOH (aq) (pre-bubbled with nitrogen) were placed in an oxygen free mini-bombs. The bombs were heated at 165 ^o^C for 3 h. Ethyl vanillin was then added to the samples and acidified with concentrated HCl. Samples were then extracted with ethyl acetate three times and dried. Lignin phenols were quantified by gas chromatography coupled with flame ion detector (GC-FID, Agilent 6890N) in Xiamen University. Standards of individual lignin phenols were purchased from Sigma-Aldrich to main the quality control of the measurement. Eight lignin phenols, vaniilin, acetovanillone, vanillic acid, syringealdehyde, acetosyringone, syringic acid, *p*-hydroxycinnamic acid and ferulic acid were quantified. The analytical error for the total eight lignin concentration is better than 6% (average standard deviation of six groups of duplicate or triplicate samples).

### Calculation of lignin flux

To calculate the constitutes flux, the relationship between different constitutes discharge (e.g., lignin phenols) and water discharge was fit by the power relationship as follows^4^,^50^:


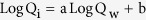


Where Q_i_ was the discharge of constitute, which was calculated by multiplying discharge with concentration, Q_w_ was the discharge, a and b are the fitting coefficient. The flux of constitutes was then calculated by putting the continuous record of water discharge in above equation.

## Additional Information

**How to cite this article**: Bao, H. *et al.* Importance of Oceanian small mountainous rivers (SMRs) in global land-to-ocean output of lignin and modern biospheric carbon. *Sci. Rep.* **5**, 16217; doi: 10.1038/srep16217 (2015).

## Supplementary Material

Supplementary Information

## Figures and Tables

**Figure 1:**
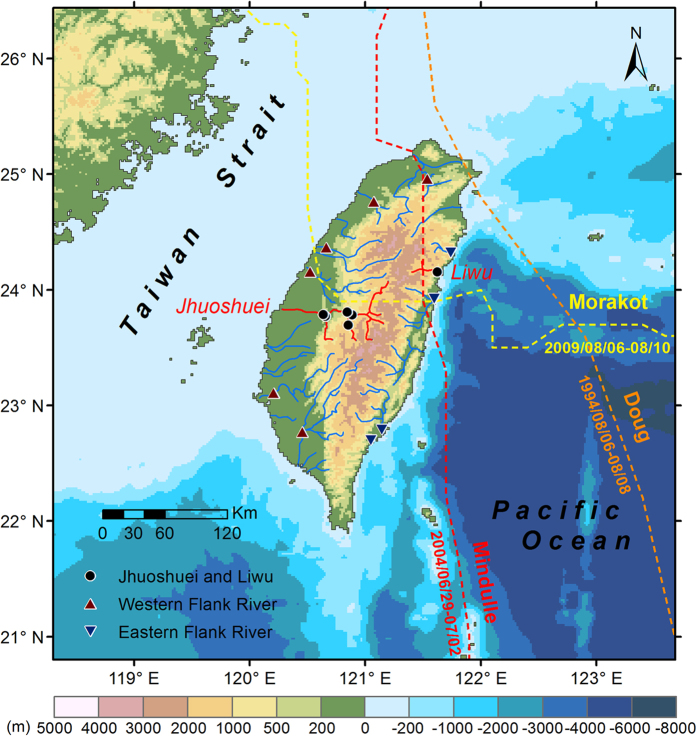
Sampling locations and tracks of Typhoon Doug (1994), Typhoon Mindulle (2004) and Typhoon Morakot (2009). The Central Mountain Range separates the island into western and eastern parts. Western-flank rivers were sampled during Doug and eastern rivers during Morakot. The map was created by the ArcGIS 10.2. The typhoon tracks were downloaded from http://www.wz121.com/TyphoonWeb/HistoryTyphoon.aspx.

**Table 1:** Lignin flux and areal lignin yield from global rivers.

River name	Basin Area(km^2^)	POC flux(Tg yr^−1^)	Lignin flux(Gg yr^−1^)	Lignin yield*(t km^−2^ yr^−1^)
Taiwan SMRs^a^	35980	1.60	19.7	0.55
Oceanian SMRs^b^	2700000	48.00	1476	0.55
Amazon River^c^	7050000	13.00	291.0	0.04
Changjiang^d^	1940000	0.99	14.9	0.01
Mississippi River^e^	3270000	0.93	13.0	0.00
Mekong River^f^	795000	1.70	19.0	0.02
Congo River^g^	3820000	2.80	87.3	0.02
Arctic rivers^h^	11103000	1.20	4.7	0.00
Eel River^i^	9537	0.01–0.02	0.11–0.29	0.01–0.04
Umpqua River^i^	13000	0.01	0.38–0.40	0.03–0.04
Total	30700537	68.6	1899	

^*^Lignin yield = lignin flux/basin area.

^a^Kao *et al.*, 2014^17^ and this study.

^b^Data are from Lyons *et al.*, 2002^10^; Kao *et al.*, 2014^17^ and this study.

^c^Data are from Hedges *et al.*, 1986^51^; Richey *et al.*, 1990^56^.

^d^Data are from Dagg *et al.*, 2004^57^; Yu *et al.*, 2011^40^; Gao *et al.*, 2012^58^.

^e^Data are from Dagg *et al.*, 2004^57^; Bianchi *et al.*, 2007^41^.

^f^Data from Ellis *et al.* (2012)^53^;

^g^Data are from Dagg *et al.*, 2004^57^; Spencer *et al.*, 2012^52^.

^h^Data from Lobbes *et al.* (2000)^59^.

^i^Data from Goñi *et al.* (2013)^6^.
