# Erratum: Importance of Oceanian small mountainous rivers (SMRs) in global land-to-ocean output of lignin and modern biospheric carbon

**DOI:** 10.1038/srep20710

**Published:** 2016-02-12

**Authors:** Hongyan Bao, Tsung-Yu Lee, Jr-Chuan Huang, Xiaojuan Feng, Minhan Dai, Shuh-Ji Kao

Scientific Reports 5: Article number: 16217; 10.1038/srep16217 published online: 11202015; updated: 02122016.

This article contains a typographical error in the ‘Introduction’ section.

“These small mountainous watersheds were rapidly flushed during typhoons, the lag time between flood peak and rain peak is only ~6 hours^12,40^…”

should read:

“These small mountainous watersheds were rapidly flushed during typhoons, the lag time between flood peak and rain peak is only ~6 hours^12,37^…”

Additionally, this article contains a typographical error in the ‘Results and Discussion’ section.

“Since the lag time between rain peak and flood peak is within a day^12,40^…”

should read:

“Since the lag time between rain peak and flood peak is within a day^12,37^…”

