# Supplementary material for: Importance of Oceanian small mountainous rivers (SMRs) in global land-to-ocean output of lignin and modern biospheric carbon
Source: Sci Rep. 2015 Nov 20;5:16217. doi: 10.1038/srep16217 (PMC4653641; doi:10.1038/srep16217)
Supplement: Supplementary Information [file srep16217-s1.pdf]

Supplementary information for the manuscript:

Importance of Oceanian small mountainous rivers (SMRs) in global land-to-ocean output of lignin and modern biospheric carbon

Hong-yan Bao[1], Tsung-Yu Lee[2], Jr-Chuan Huang[3], Xiao-juan Feng[4], Min-han Dai[1], Shuh-Ji Kao[1,*]

[1]State Key Laboratory of Marine Environmental Science, Xiamen University, 361102, Xiamen, China

[2]Department of Geography, National Taiwan Normal University, Taipei, Taiwan

[3]Department of Geography, National Taiwan University, Taipei, Taiwan

[4]State Key Laboratory of Vegetation and Environmental Change, Institute of Botany, Chinese Academy of Science, Beijing, China

[*]Corresponding author: sjkao@xmu.edu.cn

Tel: +86-592-2880178

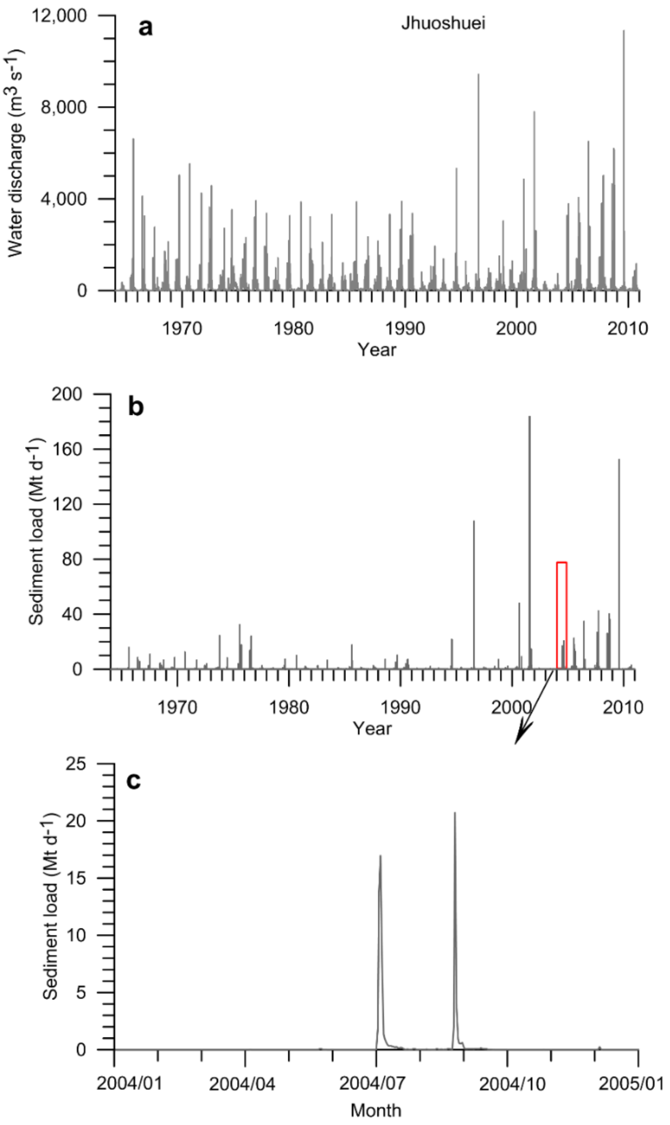

Figure S1: The historical data of Jhuoshuei River for (**a**) daily water discharge and (**b**) sediment load. The data prior to 2003 was published previously (Kao and Milliman, 2008). Here we update the data set to 2010 to include the two mentioned typhoon events. (**c**) Blow-up of the daily sediment load in 2004. As shown in the figure, the annual sediment load is completely controlled by event peaks.

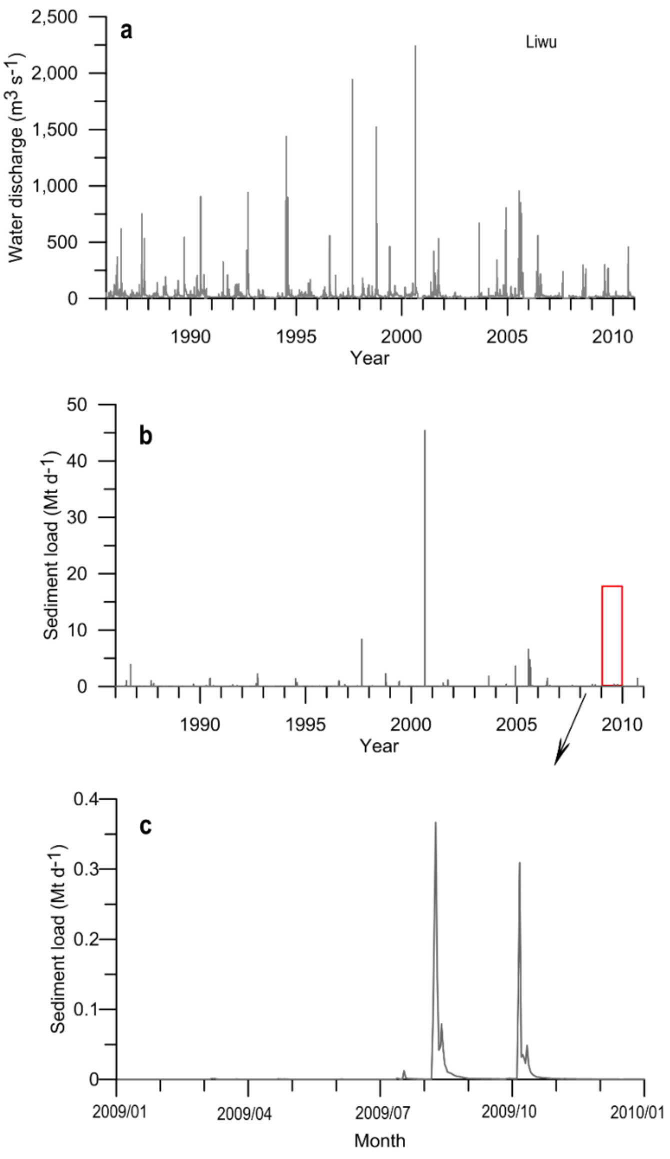

Figure S2: The historical data of Liwu River for (**a**) daily water discharge and (**b**) sediment load. (**c**) Blow-up for daily sediment load in 2009. As shown in b, the daily sediment load during our sampling, unfortunately, was < 1% of the maximum peak discharge in the record of 1986-2010.

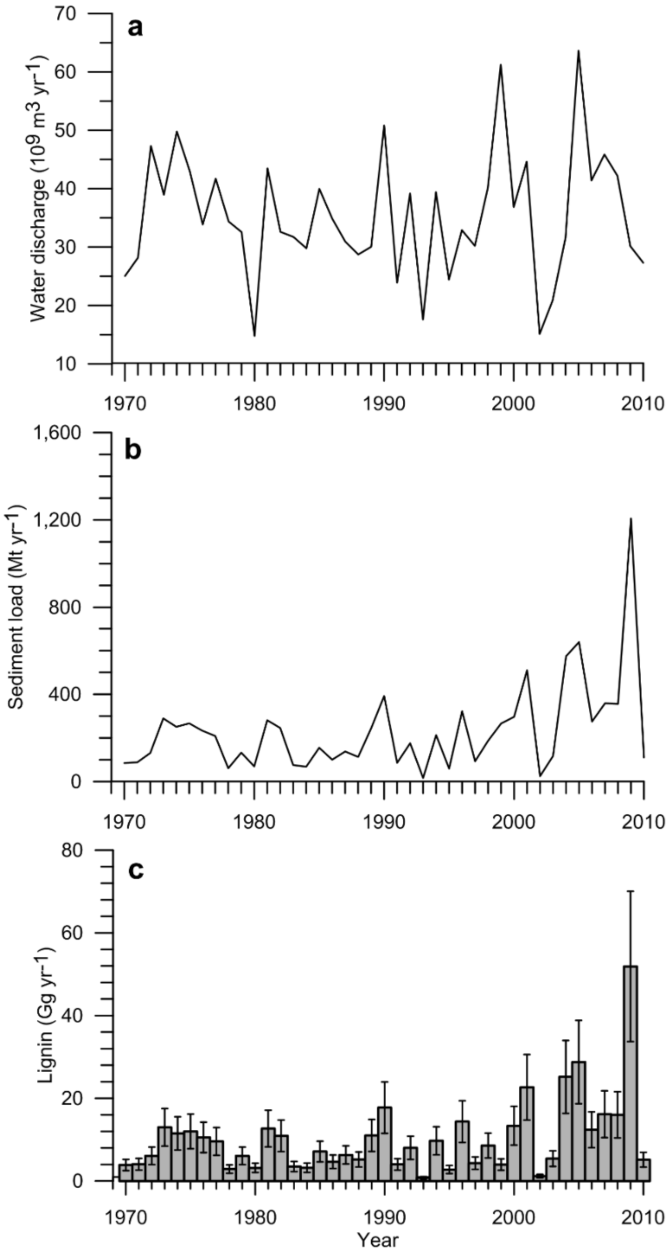

Figure S3: The historical data for (**a**) annual total water discharge, (**b**) sediment load, and (**c**) lignin from 16 Taiwan Rivers (total basin area is 18,721 km^2, approximately half of Taiwan Island). The annual lignin flux is calculated by the rating curve shown in Fig. DR3. Error bar in (**c**) is derived by residual error in Fig. 4. The average areal yield of lignin is 0.55 t km^2 yr^{-1} for 16 watersheds.

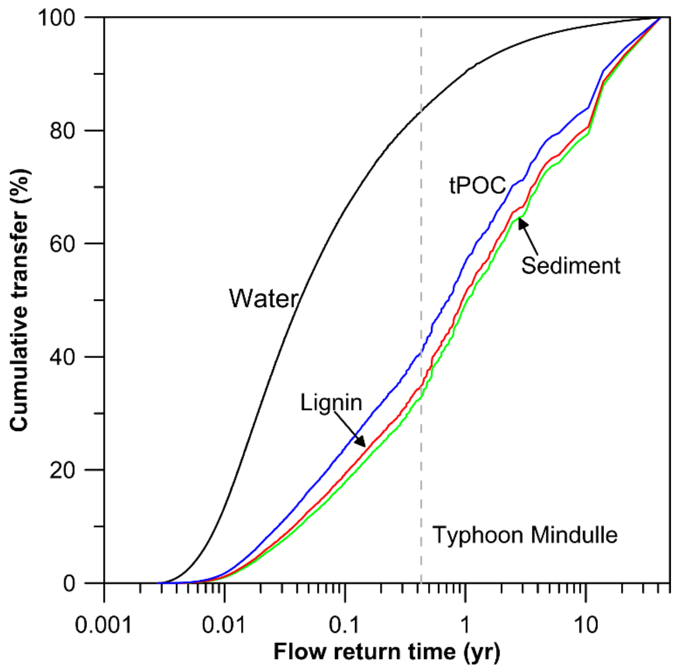

Supplementary Fig. S4: Cumulative transfer of water, sediment, lignin and tPOC versus flow return time. The flow return time is calculated according to Hitlon et al. (2008). tPOC export is calculated based on the rating curve shown in supplementary Fig. S5.

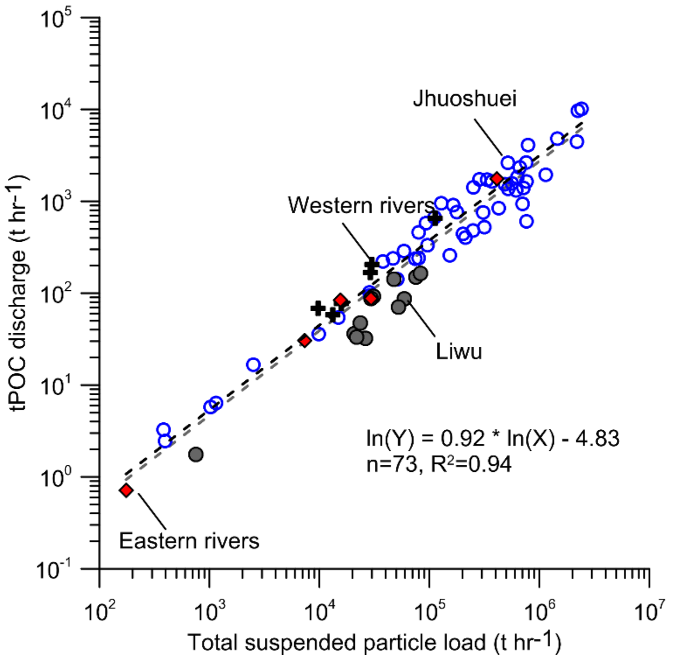

Supplementary Figure S5: Power relationship between Total suspended particle load (t hr^{-1}) and POC discharge (t hr^{-1}). The gray regression stands for the original log-log linear regression. The black dashed line and the equation are the bias-corrected for back transformation log-log linear regression following Kao et al. (2005). The residual is 19% for both over- and under-estimate.

Table S1: Geomorphic characteristics, sediment yield, mean runoff of the sampled rivers. Data sources: [*] Data from Kao and Milliman (2008); [a] Data from Yeh et al. (2007); [b]Data from Hilton et al. (2012).

River name	Latitude (ºN)	Longitude (ºE)	Length (km)	Basin Area (km^2)	Maximum elevation (m)	Mean gradient (%)	Mean runoff ($mm\ yr^{-1}$)	Mean Sediment load ($Mt\ yr^{-1}$)
Jhuoshuei[*]	120.636	23.788	186	2975	3416	1.8	1200	40±5.7
Dajia[*]	121.536	24.958	140	1292	3639	2.6	2000	0.5±0.2
Zengwun[*]	120.205	23.108	138	988	2440	1.8	900	12±2.4
Toucian[*]	121.075	24.760	63	499	2233	4.4	1400	1.1±0.2
Daan[*]	120.664	24.366	96	756	3296	3.4	1500	4.0±0.6
Wu[*]	120.522	24.154	117	1981	2596	2.2	1800	5.3±1.5
Gaoping[*]	120.454	22.770	171	3076	3997	2.3	2400	20±3.0
Hoping[*]	121.742	24.324	101	553	3742	3.7	2100	15±4.1
Zhiben[a]	121.051	22.698	39	198	2735	7	1037	N.A.
Siouguluan[*]	121.597	23.924	81	1539	2360	2.9	2200	13±1.8
Beinan[*]	121.144	22.792	83	1584	3666	4.4	1900	20±2.9
LiWu[b]	121.622	24.156	55	616	3449	6.3	2175	19

Table S2: Basic information for 16 Taiwanese rivers and the record periods of Water Resources Agency (WRA) data. Data sources: * Kao and Milliman (2008).

River	Basin Area* (km^2)	Mean annual runoff * ($mm\ yr^{-1}$)	Record period
Toucian	499	1400	1951-2010
Houlong	472	1300	1981-2010
Daan	633	1500	1966-2002,2003-2010
Dajia	916	2000	1979-2003
Wu	1981	1800	1966-2010
Jhuoshuei	2975	1200	1964-2010
Beigang	597	1300	1949-2010
Bajhang	441	1500	1960-2009
Zengwun	988	900	1960-2010
Erren	140	1700	1971-2010
Gaoping	3076	2400	1951-2010
Beinan	1584	1900	1948-2003,2005-2010
Siouguluan	1539	2200	1969-2010
Hualien	1506	2100	1969-2010
Hoping	553	2100	1975-2008
Lanyang	821	2400	1949-2010

References:

Hilton, R. G., Galy, A., Hovius, N., Chen, M. C., Horng, M. J. and Chen, H., 2008, Tropical-cyclone-driven erosion of the terrestrial biosphere from mountains: Nature Geoscience, v. 1, p. 759–762.

Hilton, R.G., Galy, A., Hovius, N., Kao, S.-J., Horng, M.-J., and Chen, H., 2012, Climatic and geomorphic controls on the erosion of terrestrial biomass from subtropical mountain forest: Global Biogeochemical Cycles, v. 26, doi: 10.1029/2012GB004314.

Kao, S., Lee, T.-Y., and Milliman, J.D., 2005, Calculating Highly Fluctuated Suspended Sediment Fluxes from Mountainous Rivers in Taiwan: TAO, v. 16, no. 3, p. 653–675.

Kao, S.J., and Milliman, J.D., 2008, Water and Sediment Discharge from Small Mountainous Rivers, Taiwan: The Roles of Lithology, Episodic Events, and Human Activities: The Journal of Geology, v. 116, p. 431–448, doi: 10.1086/590921.

Yeh, H.F., Lee, C.H., Hsu, K.C., Chen, C.T., Chang, P.H., Chen, W.C., and Tsai, D.T., 2007, Investigation of Hydrogeology and Environmental Geology in Chih-Pen Creek Basin: Paper Presented at 2007 CIMME Annual Convention, October 26th, 2007, Kaohsiung, p. 120–129.
